# Spectroscopic MRI-Guided Proton Therapy in Non-Enhancing Pediatric High-Grade Glioma

**DOI:** 10.3390/tomography9020051

**Published:** 2023-03-09

**Authors:** Vicki Huang, Abinand Rejimon, Kartik Reddy, Anuradha G. Trivedi, Karthik K. Ramesh, Alexander S. Giuffrida, Robert Muiruri, Hyunsuk Shim, Bree R. Eaton

**Affiliations:** 1 Department of Radiation Oncology, Emory University School of Medicine, Atlanta, GA 30322, USA; 2 Department of Biomedical Engineering, Emory University and Georgia Institute of Technology, Atlanta, GA 30332, USA; 3 Department of Radiology and Imaging Sciences, Emory University School of Medicine, Atlanta, GA 30322, USA; 4 Department of Radiology, Children’s Healthcare of Atlanta, Atlanta, GA 30342, USA; 5 Winship Cancer Institute, Emory University School of Medicine, Atlanta, GA 30322, USA

**Keywords:** spectroscopic MRI, proton therapy, pediatric high-grade glioma

## Abstract

Radiation therapy (RT) is a critical part of definitive therapy for pediatric high-grade glioma (pHGG). RT is designed to treat residual tumor defined on conventional MRI (cMRI), though pHGG lesions may be ill-characterized on standard imaging. Spectroscopic MRI (sMRI) measures endogenous metabolite concentrations in the brain, and Choline (Cho)/N-acetylaspartate (NAA) ratio is a highly sensitive biomarker for metabolically active tumor. We provide a preliminary report of our study introducing a novel treatment approach of whole brain sMRI-guided proton therapy for pHGG. An observational cohort (c1 = 10 patients) receives standard of care RT; a therapeutic cohort (c2 = 15 patients) receives sMRI-guided proton RT. All patients undergo cMRI and sMRI, a high-resolution 3D whole-brain echo-planar spectroscopic imaging (EPSI) sequence (interpolated resolution of 12 µL) prior to RT and at several follow-up timepoints integrated into diagnostic scans. Treatment volumes are defined by cMRI for c1 and by cMRI and Cho/NAA ≥ 2x for c2. A longitudinal imaging database is used to quantify changes in lesion and metabolite volumes. Four subjects have been enrolled (c1 = 1/c2 = 3) with sMRI imaging follow-up of 4–18 months. Preliminary data suggest sMRI improves identification of pHGG infiltration based on abnormal metabolic activity, and using proton therapy to target sMRI-defined high-risk regions is safe and feasible.

## 1. Introduction

Pediatric high-grade glioma (pHGG) is a highly aggressive disease with a 3-year overall survival of less than 30% and is responsible for over 40% of childhood brain tumor deaths [1,2]. While there is overlap in imaging and histologic features between pHGG and adult HGG, pHGG present distinct genetic and molecular differences that have hampered the success of novel therapeutic treatments based on adult HGG research [3,4]. The current standard of care for pHGG treatment utilizes a multimodal approach of surgery, radiation therapy (RT), and chemotherapy [3,5]. Although RT has been shown to significantly increase survival and is essential in maintaining effective long-term control, it has also been associated with treatment-related toxicities. With increased survival outcomes, long-term comorbidities can especially impact quality of life in the vulnerable pediatric population as they may contribute to the development of secondary cancer, neuro-psychological disorders, growth retardation, and poor social adjustment. Furthermore, multiple studies have correlated RT-related toxicities with substantial neuro-cognitive functional impairment [6,7,8,9].

Proton therapy has significant dosimetric advantages compared to photon RT. Previous studies applying proton therapy to the pediatric population have shown decreased risk for toxicity by lowering irradiation of healthy organs while still targeting the tumor [10,11,12,13,14]. Current standard of care photon RT in pHGG includes targeting the residual gross disease and/or resection cavities as defined by contrast enhancing T1-weighted (T1w-CE) MRI with a high dose (60 Gy) and T2-weighted fluid attenuated inversion recovery (T2 FLAIR) MRI with a lower dose (51–54 Gy) with a homogenous margin expansion of 5–15 mm in all directions. This technique is suboptimal as pHGG lesions may be non-contrast enhancing and diffuse with indistinct borders making them ill-characterized in standard imaging. Furthermore, tumor margins are difficult to define due to post-operative changes, heterogeneous textures, and lesion irregularity as tumors do not infiltrate homogenously. There is a critical unmet need for better detection of tumor margins to avoid exclusion of viable tumor tissue when RT targets are planned. 

Spectroscopic MRI (sMRI) is a non-contrast imaging technique measuring endogenous metabolite concentrations within tissue and can identify regions of high-risk brain tumor beyond anatomical MRI. N-acetylaspartate (NAA) is a biomarker for normal, healthy neurons that has been shown to decrease in tumors due to the replacement of normal neuronal elements by tumor cells [15,16]. Choline (Cho) is found in cell membranes and typically will be increased in regions of tumor cell proliferation. Due to the reduction of NAA and an increase in Cho, sMRI provides a highly sensitive biomarker for regions of metabolically active tumor via characterization of the Cho/NAA ratio [17,18]. Our group’s recent multi-institutional trial using sMRI to guide high-dose RT in adult glioblastoma (GBM) patients demonstrated a 7-month prolonged overall survival compared to standard RT control guided by standard MRI [19]. While the benefits have been documented in adult GBM, sMRI has yet to be applied to pediatric glioma patients. This study aims to combine the technical advantages of sMRI with the favorable outcomes of proton therapy in a pediatric study using sMRI to guide the RT treatment target. For the first time, sMRI is now integrated directly into the clinical workflow allowing its use not only for RT planning but also for the assessment of long-term treatment response as well. Design, methodology, and two preliminary patient cases with non-enhancing tumor are shown with a longitudinal follow-up. Applying sMRI to pHGG is an exciting and innovative approach to improve glioma detection and RT planning. 

## 2. Materials and Methods

This study was conducted at Emory University (Atlanta, GA, USA) and approved by the institutional review board. The study was registered with the National Clinical Trials Network (NCT04908709).

### 2.1. Study Design

**Figure 1:**
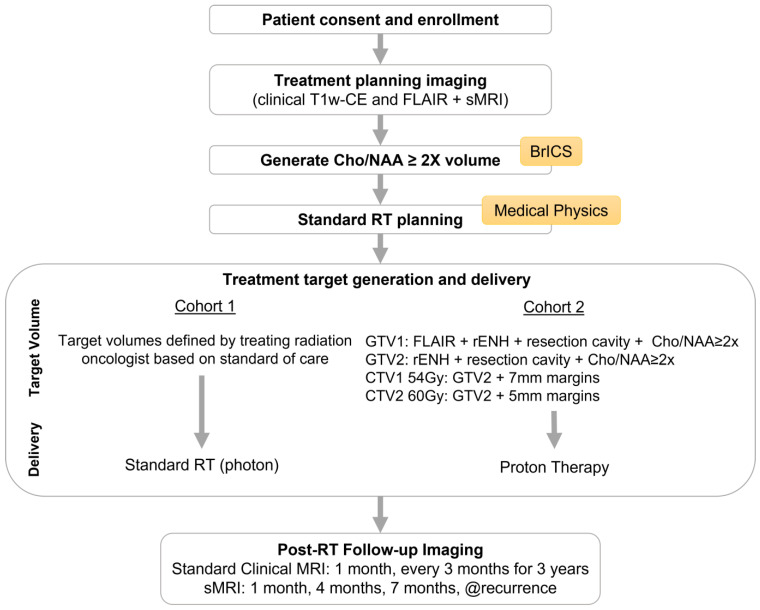
Flowchart of study design. Following consent and enrollment, all patients receive cMRI and sMRI. Their imaging data are processed and edited in BrICS to generate sMRI target volumes before exporting to the RT planning system for execution. The target volumes for c1 are based solely on cMRI, and they receive photon RT based on standard of care. The target volumes for c2 are based on cMRI + sMRI, and they receive proton therapy. Both cohorts receive the same post-RT follow-up imaging protocol of cMRI beginning at 1 month post-RT and continuing in 3-month intervals up to 3 years with concurrent sMRI for the first three timepoints and then followed as needed or at the time of tumor recurrence.

This study evaluates the role of whole brain sMRI to guide the high-dose target of proton therapy in pHGG patients. This study includes an observational cohort (c1 = 10 patients) receiving standard of care RT and a therapeutic cohort (c2 = 15 patients) being treated using sMRI-guided proton RT. A flowchart detailing the study design is shown in Figure 1. All patients will be imaged with the same protocol consisting of standard clinical MRI (T1w-CE and T2 FLAIR) and sMRI with a high-resolution 3D whole-brain echo-planar spectroscopic imaging (EPSI) sequence. Baseline images (sMRI and standard clinical MRI) will be acquired prior to RT for treatment planning. Treatment volumes based on standard MRI will be created, and for c2 the 60 Gy high-dose target will be guided by the T1w-CE contrast-enhancing region combined with sMRI Cho/NAA ≥ 2x (metabolite ratio elevated to twice the value of the mean Cho/NAA in normal appearing white matter contralateral to the primary tumor) volume. In the absence of an enhancing tumor, subjects will be treated with a single intermediate dose level of 54 Gy guided by the summation of FLAIR abnormality with Cho/NAA ≥ 2x volume. After 6 weeks of RT, follow-up standard MRIs will be obtained beginning at 1 month post-RT completion and every 3 months afterwards for 3 years. sMRI will be acquired alongside standard imaging for the first three follow-ups (1, 4, and 7 months) and then as needed or at suspected tumor progression. Our EPSI sequence was installed and tested on the hospital scanner, which enabled seamless acquisition of the sMRI scan with clinical diagnostic MRIs beginning at the 4 month post-RT timepoint in Subject 2. Given the novelty of this study in the pediatric population, we needed to establish an infrastructure for sMRI to be performed in a clinical setting, with an easily accessible pipeline for obtaining longitudinal sMRI data via our Brain Imaging Collaboration Suite (BrICS) [20]. This infrastructure will provide the foundation necessary for this study and future studies incorporating sMRI, which require longitudinal imaging follow-up.

### 2.2. Enrollment Eligibility Criteria

Patients eligible for enrollment in this study met all eligibility criteria. The inclusion criteria were that the patient must be less than 21 years old at the time of registration, have a diagnosis of WHO grade III–IV glioma confirmed pathologically, primary tumor located within supratentorial brain, recommended to receive RT treatment, and were able to receive MRI scans. No gender, race, or ethnic criteria are required to be met. Patients were excluded if they had pacemakers, non-titanium metal surgical clips, implants, or other MRI-incompatible non-removable medical devices. Additional exclusion criteria include any significant medical illnesses that would prevent the patient from tolerating an MRI or if the tumor was demonstrated by pathology to be either not a glioma or low-grade glioma. 

### 2.3. Image Acquisitions/sMRI-Based Target Generation

Clinical imaging for this study was performed at Children’s Healthcare of Atlanta Scottish Rite Hospital. All patients enrolled underwent the standard brain tumor protocol. For the RT planning, this included a CT simulation (≤1 mm slice thickness) and a T1w-CE and T2 FLAIR MRI. The sMRI was either integrated into the same clinical imaging session as the standard sequences or obtained in a separate appointment around the same time period. The whole brain EPSI had a scan time of 14 min and was acquired with GRAPPA parallelization on Siemens 3T Prisma scanners using either a 32- or 20-channel head and neck coil (echo time [TE] = 70 ms, repetition time [TR] = 975 ms, flip angle [FA] = 71°) [21,22,23,24,25]. The scan had an FOV of 170 mm × 260 mm × 120 mm and a matrix size of 64 × 50 × 22 with a nominal voxel size of 74.34 µL. Following post-processing, the final matrix size is 85 × 130 × 40 with an interpolated resolution of 2 mm × 2 mm × 3 mm and a final voxel size of 12 µL. The sequence had no inversion recovery for fat suppression allowing for a shortened TR to ensure a clinically acceptable scan time while also achieving 9x smaller interpolated voxel size than that in our previously reported adult GBM study [18,19]. During the same imaging session as the sMRI, a pre-contrast T1w MRI with 1 mm isotropic resolution was acquired. Data were transferred and processed offline using the MIDAS software suite (University of Miami, Miami, FL, USA)/BrICS (Emory) to create co-registered spatial–spectral data and normalized metabolite ratio maps overlaid on clinical images [20,26]. For spectral quality assurance, MIDAS includes two preset filters, which eliminate poor spectra with linewidth greater than 12 Hz for water T2 or 13 Hz for metabolic spectra.

The sMRI-guided high-dose target volumes were generated using high-resolution color maps demonstrating Cho/NAA ≥ 2x abnormality co-registered to high-resolution structural MRI sequences in BrICS, our customized cloud platform for integrating sMRI with RT planning [20]. Images were first sent in DICOM format to a localized HIPAA-compliant server and uploaded into BrICS. All anatomical images, single metabolite maps, and metabolite ratio maps were interpolated and co-registered to a single coordinate system based on the high-resolution isotropic T1w MRI. Contours at the Cho/NAA ≥ 2x threshold were generated in BrICS [18,20]. The residual contrast-enhancing (rENH) volumes on the T1w-CE MRI excluded post-surgical related enhancing regions. Segmentation volumes were reviewed and manually edited by an MR spectroscopy expert and pediatric neuroradiologist and subsequently exported from BrICS in the DICOM format and imported into the RT planning system for execution (Eclipse/VelocityAI, Varian Medical Systems).

### 2.4. RT Planning

Pre-operative and post-operative diagnostic MRIs are co-registered to the CT simulation to provide improved resolution for target and normal structure identification. Gross tumor volumes (GTV) are defined by the treating radiation oncologist based solely on the conventional MRI (cMRI) prior to viewing the sMRI for both cohorts. For pHGG with a contrast enhancing tumor, the GTV2 is defined as the resection cavity and any residual contrast-enhancing disease. In c2 only, the sMRI Cho/NAA ≥ 2x volume is added to the GTV2. The GTV1 includes GTV2 and any adjacent T2/FLAIR signal abnormality. The corresponding clinical target volumes (CTV1 and CTV2) are defined using a 5 mm and 7 mm anatomically modified expansion margin from GTV1 and GTV2, respectively. CTV1 is prescribed 54 Gy (RBE) in 1.8 Gy per fraction and CTV2 is prescribed 60 Gy (RBE) in 2 Gy per fraction using a simultaneous integrated boost. 

In the setting of non-enhancing diffuse disease with infiltrative involvement or a supratentorial diffuse midline glioma, no CTV2 is defined, and the cumulative prescription dose is 54 Gy (RBE). No planning target volume (PTV) margin was used for proton therapy. All proton therapy treatment employs pencil beam scanning prescribed to the CTV with robust optimization using a ±3 mm robustness margin and ±3.5% range uncertainty.

Standard photon RT plans using a 3 mm PTV margin expansion and volumetric modulated arc therapy (VMAT) are generated for both cohorts for comparative data analysis of target volume and normal tissue dose statistics.

### 2.5. RT Treatment Delivery

All enrolled patients received RT treatment in 30 fractions over 6 weeks. Patients were seen by the treatment radiation oncologist weekly for history and physical exam, and all adverse events were prospectively recorded, graded according to the Common Terminology for Adverse Events (CTCAE v5.0), and assigned an attribution score of either unrelated, unlikely related, possibly related, probably related, or definitively related to RT. Chemotherapy was not required but may be prescribed at the discretion of the treating pediatric neuro-oncologist.

### 2.6. Longitudinal Imaging Follow-Up

Follow-up MRIs are obtained 1 month post-RT and continued every 3 months afterwards for 3 years, or until unequivocal progression or the patient is removed from the study. sMRI will be acquired alongside standard imaging for the first three time points (1, 4, and 7 months) and then as needed or at suspected tumor recurrence. A longitudinal database was built to quantify treatment response through lesion and metabolite volumes. Each follow-up was uploaded into the BrICS Longitudinal Image Tracker (BrICS-LIT) module for analysis along with RT dose plans [27]. Regions of tumor progression were determined by a multi-disciplinary translational research team consisting of a pediatric radiation oncologist, pediatric neuroradiologist, and MR spectroscopy experts. The T1w-CE and T2 FLAIR images were contoured using the semi-automated algorithm in BrICS-LIT prior to being manually adjusted by an experienced pediatric neuroradiologist. Each time point was analyzed and scored according to criteria defined by the Brain Tumor Reporting and Data System (BT-RADS) [28,29,30,31,32]. At the time of progression, recurrent volumes are contoured and compared to the initial GTV and radiation dose distribution to be scored as either local (in-field), marginal (immediately outside margin-of-field), or distant (away from margin-of-field) recurrences.

## 3. Results

We have currently enrolled 4 subjects (c1 = 1/c2 = 3) with imaging timepoints spanning 4 to 18 months. This study presents initial observations comparing differences between target treatment volumes determined by cMRI and sMRI, as well as their corresponding longitudinal treatment response. Using our ultra-high-resolution EPSI sequence, the average brain coverage across all subjects is 80.14%. Three patients presented tumor without enhancement and were treated to a maximum intermediate dose of 54 Gy, and one patient in c2 was treated with a high-dose target of 60 Gy. The median difference between the GTV1 cMRI only and GTV1 with Cho/NAA ≥ 2x guidance is 10.68 cc.

### 3.1. Subject 1

**Figure 2:**
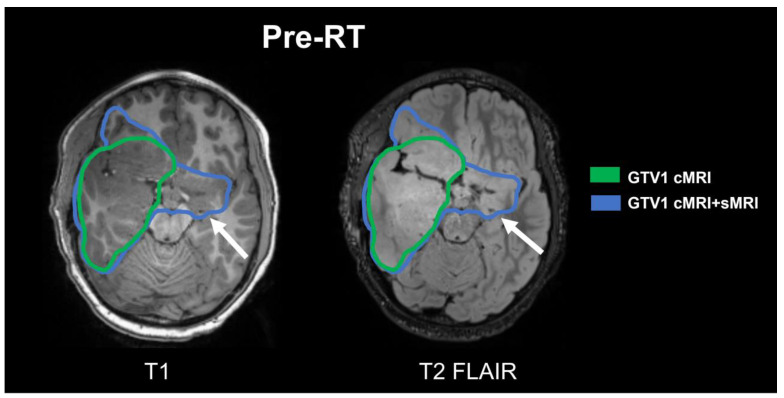
Comparison of GTV1 contours. Target volumes based on cMRI alone (green) and cMRI + sMRI (blue) are shown overlaid on axial slices of the T1 (**left**) and T2 FLAIR (**right**) images. The sMRI detected an additional lesion in the left temporal lobe which is non-hyperintense on FLAIR and absent on the T1 imaging as well (white arrow). The volume of GTV1 cMRI was 207.61 cc compared to the GTV1 cMRI + sMRI volume of 297.84 cc.

**Figure 3:**
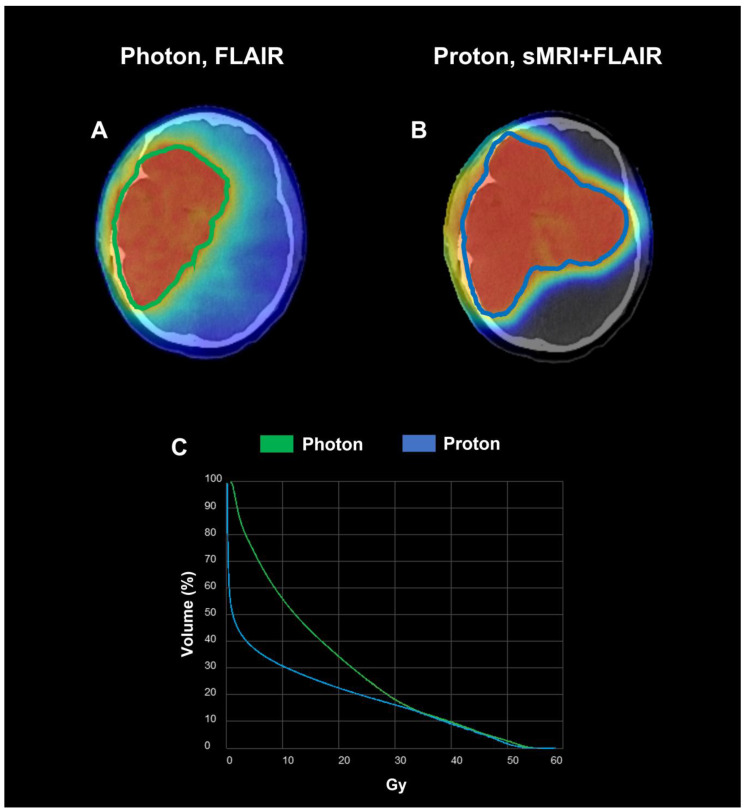
Comparison of photon (VMAT) and proton dose plans. The photon plan was created using cMRI with the target volume (green contour) based on the FLAIR (**A**). The proton plan was based on sMRI (Cho/NAA ≥ 2x) and FLAIR with target volume (blue contour) corresponding to the union of the sMRI + FLAIR abnormality (**B**). Both targets show volume treated to 54 Gy intermediate dose due to lack of enhancing tumor on T1w-CE. Heat map shows 54 Gy area in orange with a larger target volume for tumor and much less scattering radiation to normal tissue in the proton plan versus the photon plan as shown in the brain dose volume histogram graph (**C**).

The first subject is a 9-year-old male with IDH wild-type WHO Grade III diffuse HGG with anaplastic astrocytoma morphology in the right temporal lobe; he was enrolled in the trial 1 month after resection. Initial pre-RT Cho/NAA ≥ 2x volume was 165.00 cc compared to FLAIR volume of 207.21 cc including an additional region in the left temporal lobe, which is non-hyperintense on FLAIR. The sMRI changed the treatment plan significantly by revealing the presence of disease infiltration to the contralateral brain previously not detected on cMRI (Figure 2). The resultant volume for GTV1 cMRI + sMRI was 297.84 cc compared to 207.21 cc from GTV1 cMRI. The subject received 54 Gy (RBE) proton therapy to GTV1 cMRI + sMRI. After a follow-up period of 18 months, the most recent imaging time point was determined as tumor progression (15 months post-RT) both local and distant to the RT field. The patient’s proton RT plan is shown in Figure 3 alongside a comparative standard of care photon RT with a corresponding dose–volume histogram. 

**Figure 4:**
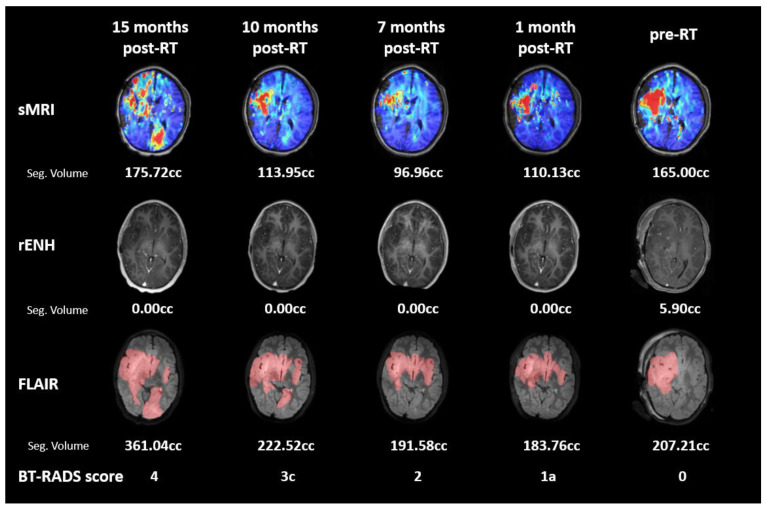
Subject 1 longitudinal follow-up imaging shown in BrICS-LIT. Five follow-up images and their corresponding sMRI, T1w-CE, and T2 FLAIR are shown chronologically from right to left, with the pre-RT treatment planning image first. Lesion volumes have been segmented, and each follow-up is scored by a neuroradiologist. No contrast enhancing tumor was seen in follow-ups. The imaging shows a positive response to treatment with decreasing volumes on sMRI from pre-RT to 7 months post-RT; the FLAIR volumes decreased and remained stable during this time period. Beginning at 10 months post-RT both sMRI and FLAIR volumes began to increase, with a greater than 25% volumetric increase from 10 months to 15 months post-RT. An out-of-field metabolically active mass is present on the 15 months post-RT sMRI in the left occipital lobe.

All available follow-up time points are shown in Figure 4 in the BrICS-LIT interface. The clinical imaging shows initial positive response to treatment and then gradual disease progression. The tumor remained non-enhancing on clinical imaging throughout follow-up. T2 FLAIR volumes decreased and remained stable with less than 5% volumetric increase between 1 and 7 months post-RT. Between 10 and 15 months, there was a greater than 25% increase in T2 FLAIR volumes, and the 15 month follow-up was the officially determined date of progression (BT-RADS Score 4). The sMRI follows a similar trend with Cho/NAA ≥ 2x volumes decreasing 40% from pre-RT to 7 months post-RT, then increasing greater than 25% across the subsequent 10 and 15 months post-RT follow-up dates. 

**Figure 5:**
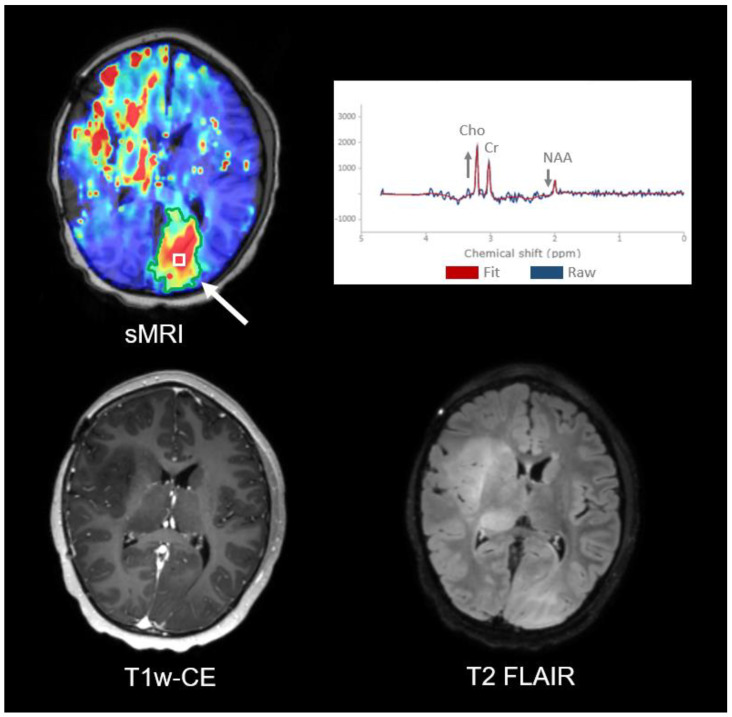
Out-of-field recurrence in Subject 1. Imaging of the sMRI, T1w-CE, and T2 FLAIR at the recurrence date (15 months post-RT) with the site of tumor progression indicated by the white arrow on sMRI. The green contour shows the additional 30.50 cc metabolically active mass at the Cho/NAA ≥ 2x threshold on the sMRI. The spectra identified by the boxed ROI on the sMRI map shows clearly elevated Cho and decreased NAA (gray arrows), corresponding to presence of tumor. The lesion is not identifiable with confidence on the T1w-CE and is better seen on sMRI than T2 FLAIR.

Out-of-field recurrence in the left occipital lobe was identified at the 15 months post-RT progression date. There is a clear metabolically active mass (30.50 cc) encompassed within the Cho/NAA ≥ 2x volume (Figure 5). Signs of this lesion first appeared on the 10 months post-RT sMRI, but it did not meet the 2x threshold. This lesion is not identifiable on the T1w-CE, and diffuse hyperintensity on the T2 FLAIR is shown. The treated area shows NAA and Cho/NAA ratio variation, possibly due to increased lipids from treatment effects. 

### 3.2. Subject 2

**Figure 6:**
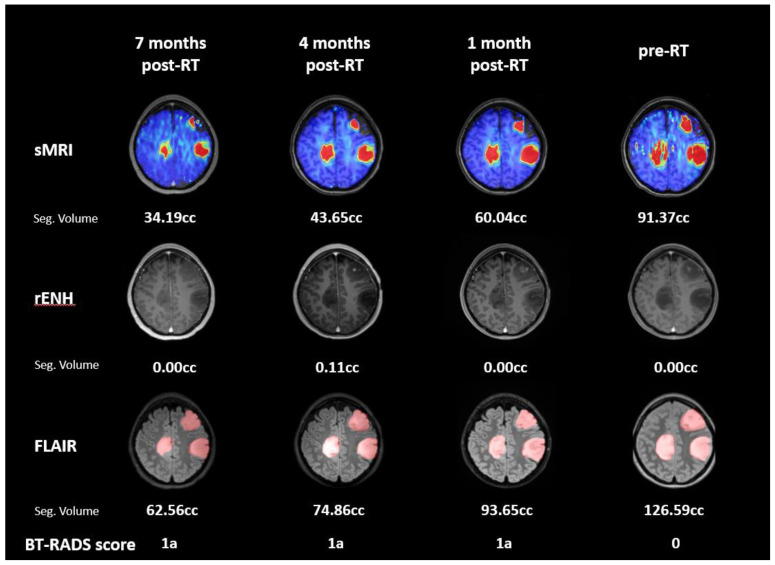
Subject 2 longitudinal follow-up imaging shown in BrICS-LIT. Four follow-up images and their corresponding sMRI, T1w-CE, and T2 FLAIR are shown chronologically from right to left, with the pre-RT treatment planning image first. Lesion volumes have been segmented, and each follow-up is scored by a neuroradiologist. Little to no contrast enhancing tumor was seen in follow-ups. The imaging shows a positive response to treatment with decreasing volumes on sMRI from pre-RT to 7 months post-RT; the FLAIR volumes also decreased during this time period. Each follow-up is scored as BT-RADS 1a corresponding to disease improvement.

The second subject is a 19-year-old female diagnosed with multi-focal IDH mutant WHO Grade III anaplastic astrocytoma; she was enrolled in the trial 1 month after biopsy and received 54 Gy proton therapy. To date, the subject has a follow-up period of 10 months (7 months post-RT). The pre-RT Cho/NAA ≥ 2x volume was 91.37 cc, and the tumor was non-enhancing. The GTV1 cMRI (FLAIR) volume was 126.59 cc, and the GTV1 cMRI + sMRI volume was 130.78 cc. Four longitudinal time points are shown in Figure 6. Lesion size in the sMRI and FLAIR volumes consistently decreased, and the Cho/NAA ≥ 2x volume agreed well with the FLAIR abnormality. Each follow-up is scored as BT-RADS 1a corresponding to improvement in disease.

## 4. Discussion

pHGG are highly aggressive brain tumors that have become the leading cause of cancer-related death in the pediatric population due to the limited efficacy of currently available treatment options [33,34,35]. This is the first description of a study design in pHGG patients, which combines the high specificity of both whole-brain high-resolution sMRI tumor detection with precise proton therapy treatment targeting. The primary objective is to determine the feasibility of sMRI-guided proton RT and to determine the value of sMRI to predict regions of brain at high risk for tumor recurrence. This trial has implemented a clinically translatable treatment pipeline by integrating sMRI into the standard clinical workflow. The single 14-minute whole-brain sMRI sequence was added into the standard brain tumor imaging protocol for planning and follow-up. Our new ultra-high-resolution EPSI sequence increased resolution from our previous study from a nominal voxel size of 314 µL to 74 µL with the final interpolated resolution decreasing from a 108 µL to 12 µL voxel size and average brain coverage across subjects of 80.14%. This sequence appeared more optimal in younger patients as the differences in water and lipid composition in pediatric compared to the adult brain may reduce artefactual lipid contribution in the sMRI data [19,36]. After acquisition, data from the sMRI are processed and available in the RT planning systems with a 1.5 day turnaround time (including all quality control steps), fitting well within the standard clinical treatment timeline [20].

Each of our cases show that sMRI identifies infiltrating tumor where there is no contrast-enhancing lesion on standard MRI and the Cho/NAA ≥ 2x volume either agrees with or further reveals disease not detectable on FLAIR. Especially, as pHGG is often non-enhancing, the T1w-CE does not demonstrate the extent of disease. In both Subjects 1 and 2, the tumor is grossly non-enhancing with absent rENH volume with diffuse infiltrative disease. Even in the absence of any contrast-enhancing region, the sMRI was informative for guiding an intermediate dose target. While the sMRI-defined targets are larger than targets defined by conventional imaging, the use of proton therapy allows safe delivery of high-dose radiation to high-risk regions without increased treatment morbidity to patients. Furthermore, proton therapy treats tumor targets with greater precision, thus reducing radiation exposure in normal developing brain, which may attenuate acute and long-term toxicity effects and improve quality of life.

A longitudinal imaging database was built in the BrICS-LIT module containing all available follow-up MRIs to visualize a comprehensive disease history. Each follow-up MRI was given a BT-RADS score classifying disease state. Although BT-RADS has been successfully implemented in adult gliomas at multiple institutions with ongoing standardization for management-based structured reporting, it has yet to be adapted for pediatrics [28,30]. Therefore, in addition to the BT-RADS score, final clinical determination of pseudo-progression or tumor progression was based on multi-disciplinary group consensus following image review. As more data is accrued, scoring guidelines for pediatric brain tumor applications will be optimized; implementation of structured clinical reporting criteria in an objective manner is advantageous for informing outcome measurements of clinical trial datasets. Additionally, the BrICS-LIT database identifies failure patterns with relation to radiation field by displaying an overlay of the dose map on top of the anatomic MRI. The database entry for Subject 1 shows gradually worsening imaging before progression was called (Figure 4). Visualization of the Cho/NAA maps for this subject alongside the clinical MRIs can prospectively identify out-of-field recurrence; early signs of elevated Cho/NAA in the left occipital lobe began as soon as 7 months post-RT. Although there is some mild T2 FLAIR hyperintensity in the same region beginning from 10 months post-RT, the abnormality is more clearly visible on sMRI generated color maps. Additionally, T2 FLAIR changes are not always tumor specific, and may be a result of numerous other etiologies, such as vasogenic edema, cytotoxic edema, treatment related changes, or artifact [37]. Our results demonstrate that elevated Cho/NAA ratio obtained with sMRI represents a novel high-resolution neuroimaging biomarker for pHGG with increased specificity over FLAIR and T1w-CE imaging.

As this is the first on-going study using sMRI to define a quantitative volume for high-dose target, the long-term treatment-related effects and outcomes are unknown. Previous studies prospectively collecting health outcomes for children treated with proton therapy defined the boosted high-dose area as post-surgical cavity combined with rENH, and they showed similar survival outcomes but significantly favorable long-term neurocognitive function compared to photon RT [12,13,14].

## 5. Conclusions

This is the first report of sMRI-guided proton therapy to treat pHGG. This methodology, combining the high specificity of sMRI with the precise targeting of proton therapy, can more specifically identify tumors and deliver effective radiation doses to a specified target while minimizing radiation scatter, lowering volumetric radiation dose in the developing brain, and potentially reducing side effects of RT for the pediatric population. Our preliminary results show that sMRI identifies metabolite changes beyond lesions shown in clinical MRI (both T1w-CE and FLAIR) and may predict sites of tumor progression. This study will further elucidate knowledge of disease control and acute effects of proton RT in children and investigates long-term metabolic changes in pediatrics.

## Data Availability

The data presented in this study are available on request from the corresponding author.

## References

[1] Jakacki R.I., Cohen K.J., Buxton A., Krailo M.D., Burger P.C., Rosenblum M.K., Brat D.J., Hamilton R.L., Eckel S.P., Zhou T. (2016). Phase 2 study of concurrent radiotherapy and temozolomide followed by temozolomide and lomustine in the treatment of children with high-grade glioma: A report of the Children’s Oncology Group ACNS0423 study. Neuro Oncol..

[2] Buccoliero A.M., Giunti L., Moscardi S., Castiglione F., Provenzano A., Sardi I., Scagnet M., Genitori L., Caporalini C. (2022). Pediatric High Grade Glioma Classification Criteria and Molecular Features of a Case Series. Genes.

[3] Thorbinson C., Kilday J.-P. (2021). Childhood Malignant Brain Tumors: Balancing the Bench and Bedside. Cancers.

[4] Mackay A., Burford A., Carvalho D., Izquierdo E., Fazal-Salom J., Taylor K.R., Bjerke L., Clarke M., Vinci M., Nandhabalan M. (2017). Integrated Molecular Meta-Analysis of 1,000 Pediatric High-Grade and Diffuse Intrinsic Pontine Glioma. Cancer Cell.

[5] Stupp R., Mason W.P., van den Bent M.J., Weller M., Fisher B., Taphoorn M.J.B., Belanger K., Brandes A.A., Marosi C., Bogdahn U. (2005). Radiotherapy plus Concomitant and Adjuvant Temozolomide for Glioblastoma. N. Engl. J. Med..

[6] Merchant T.E., Schreiber J.E., Wu S., Lukose R., Xiong X., Gajjar A. (2014). Critical Combinations of Radiation Dose and Volume Predict IQ and Academic Achievement Scores after Craniospinal Irradiation in Children with Medulloblastoma. Int. J. Radiat. Oncol. Biol. Phys..

[7] Armstrong G.T., Conklin H.M., Huang S., Srivastava D., Sanford R., Ellison D.W., Merchant T.E., Hudson M.M., Hoehn M.E., Robison L.L. (2011). Survival and long-term health and cognitive outcomes after low-grade glioma. Neuro Oncol..

[8] Oeffinger K.C., Mertens A.C., Sklar C.A., Kawashima T., Hudson M.M., Meadows A.T., Friedman D.L., Marina N., Hobbie W., Kadan-Lottick N.S. (2006). Chronic Health Conditions in Adult Survivors of Childhood Cancer. N. Engl. J. Med..

[9] Armstrong G.T., Liu Q., Yasui Y., Neglia J.P., Leisenring W., Robison L.L., Mertens A.C. (2009). Late Mortality Among 5-Year Survivors of Childhood Cancer: A Summary From the Childhood Cancer Survivor Study. J. Clin. Oncol..

[10] Mizumoto M., Fuji H., Miyachi M., Soejima T., Yamamoto T., Aibe N., Demizu Y., Iwata H., Hashimoto T., Motegi A. (2021). Proton beam therapy for children and adolescents and young adults (AYAs): JASTRO and JSPHO Guidelines. Cancer Treat. Rev..

[11] Jalali R., Goda J.S. (2019). Proton beam therapy in pediatric brain tumor patients: Improved radiation delivery techniques improve neurocognitive outcomes. Neuro Oncol..

[12] Gross J.P., Powell S., Zelko F., Hartsell W., Goldman S., Fangusaro J., Lulla R.R., Smiley N.P., Chang J.H.-C., Gondi V. (2019). Improved neuropsychological outcomes following proton therapy relative to X-ray therapy for pediatric brain tumor patients. Neuro Oncol..

[13] Yock T.I., Yeap B.Y., Ebb D.H., Weyman E., Eaton B.R., Sherry N.A., Jones R.M., MacDonald S.M., Pulsifer M.B., Lavally B. (2016). Long-term toxic effects of proton radiotherapy for paediatric medulloblastoma: A phase 2 single-arm study. Lancet Oncol..

[14] Kahalley L.S., Ris M.D., Grosshans D.R., Okcu M.F., Paulino A.C., Chintagumpala M., Moore B.D., Guffey D., Minard C.G., Stancel H.H. (2016). Comparing Intelligence Quotient Change After Treatment With Proton Versus Photon Radiation Therapy for Pediatric Brain Tumors. J. Clin. Oncol. Off. J. Am. Soc. Clin. Oncol..

[15] Laprie A., Pirzkall A., Haas-Kogan D.A., Cha S., Banerjee A., Le T.P., Lu Y., Nelson S., McKnight T.R. (2005). Longitudinal multivoxel MR spectroscopy study of pediatric diffuse brainstem gliomas treated with radiotherapy. Int. J. Radiat. Oncol. Biol. Phys..

[16] Poussaint T.Y., Rodriguez D. (2006). Advanced neuroimaging of pediatric brain tumors: MR diffusion, MR perfusion, and MR spectroscopy. Neuroimaging Clin. N. Am..

[17] Liserre R., Pinelli L., Gasparotti R. (2021). MR spectroscopy in pediatric neuroradiology. Transl. Pediatr..

[18] Cordova J.S., Shu H.-K.G., Liang Z., Gurbani S.S., Cooper L.A.D., Holder C.A., Olson J.J., Kairdolf B., Schreibmann E., Neill S.G. (2016). Whole-brain spectroscopic MRI biomarkers identify infiltrating margins in glioblastoma patients. Neuro Oncol..

[19] Ramesh K., Mellon E.A., Gurbani S.S., Weinberg B.D., Schreibmann E., Sheriff S.A., Goryawala M., de le Fuente M., Eaton B.R., Zhong J. (2022). A multi-institutional pilot clinical trial of spectroscopic MRI-guided radiation dose escalation for newly diagnosed glioblastoma. Neuro Oncol. Adv..

[20] Gurbani S., Weinberg B., Cooper L., Mellon E., Schreibmann E., Sheriff S., Maudsley A., Goryawala M., Shu H.-K., Shim H. (2019). The Brain Imaging Collaboration Suite (BrICS): A Cloud Platform for Integrating Whole-Brain Spectroscopic MRI into the Radiation Therapy Planning Workflow. Tomography.

[21] Goryawala M., Han H., Hosseini Z., Ahn S., Moran G.R., Shim H. (2020). Siemens Healthineers World Magazine: Magnetom Flash.

[22] Goryawala M., Saraf-Lavi E., Nagornaya N., Heros D., Komotar R., Maudsley A.A. (2020). The Association between Whole-Brain MR Spectroscopy and IDH Mutation Status in Gliomas. J. Neuroimaging.

[23] Goryawala M.Z., Sheriff S., Maudsley A.A. (2016). Regional Distributions of Brain Glutamate and Glutamine in Normal Subjects. NMR Biomed..

[24] Goryawala M.Z., Sheriff S., Stoyanova R., Maudsley A.A. (2018). Spectral decomposition for resolving partial volume effects in MRSI. Magn. Reson. Med..

[25] Sabati M., Sheriff S., Gu M., Wei J., Zhu H., Barker P.B., Spielman D.M., Alger J.R., Maudsley A.A. (2015). Multi-Vendor Implementation and Comparison of Volumetric Whole-Brain Echo-Planar MR Spectroscopic Imaging. Magn. Reson. Med..

[26] Maudsley A.A., Darkazanli A., Alger J.R., Hall L.O., Schuff N., Studholme C., Yu Y., Ebel A., Frew A., Goldgof D. (2006). Comprehensive processing, display and analysis for in vivo MR spectroscopic imaging. NMR Biomed..

[27] Ramesh K., Gurbani S.S., Mellon E.A., Huang V., Goryawala M., Barker P.B., Kleinberg L., Shu H.-K.G., Shim H., Weinberg B.D. (2020). The Longitudinal Imaging Tracker (BrICS-LIT):A Cloud Platform for Monitoring Treatment Response in Glioblastoma Patients. Tomogr. Ann Arbor Mich.

[28] Weinberg B.D., Gore A., Shu H.-K.G., Olson J.J., Duszak R., Voloschin A.D., Hoch M.J. (2018). Management-Based Structured Reporting of Posttreatment Glioma Response With the Brain Tumor Reporting and Data System. J. Am. Coll. Radiol..

[29] Gore A., Hoch M.J., Shu H.-K.G., Olson J.J., Voloschin A.D., Weinberg B.D. (2019). Institutional Implementation of a Structured Reporting System: Our Experience with the Brain Tumor Reporting and Data System. Acad. Radiol..

[30] Zhang J.Y., Weinberg B.D., Hu R., Saindane A., Mullins M., Allen J., Hoch M.J. (2020). Quantitative Improvement in Brain Tumor MRI Through Structured Reporting (BT-RADS). Acad. Radiol..

[31] Kim S., Hoch M.J., Peng L., Somasundaram A., Chen Z., Weinberg B.D. (2022). A brain tumor reporting and data system to optimize imaging surveillance and prognostication in high-grade gliomas. J. Neuroimaging.

[32] Kim S., Hoch M.J., Cooper M.E., Gore A., Weinberg B.D. (2021). Using a Website to Teach a Structured Reporting System, the Brain Tumor Reporting and Data System. Curr. Probl. Diagn. Radiol..

[33] Haase S., Nuñez F.M., Gauss J.C., Thompson S., Brumley E., Lowenstein P., Castro M.G. (2020). Hemispherical Pediatric High-Grade Glioma: Molecular Basis and Therapeutic Opportunities. Int. J. Mol. Sci..

[34] Ostrom Q.T., Gittleman H., Truitt G., Boscia A., Kruchko C., Barnholtz-Sloan J.S. (2018). CBTRUS Statistical Report: Primary Brain and Other Central Nervous System Tumors Diagnosed in the United States in 2011-2015. Neuro Oncol..

[35] Sturm D., Pfister S.M., Jones D.T.W. (2017). Pediatric Gliomas: Current Concepts on Diagnosis, Biology, and Clinical Management. J. Clin. Oncol..

[36] Kreis R., Ernst T., Ross B.D. (1993). Development of the human brain: In vivo quantification of metabolite and water content with proton magnetic resonance spectroscopy. Magn. Reson. Med..

[37] Villanueva-Meyer J.E., Mabray M.C., Cha S. (2017). Current Clinical Brain Tumor Imaging. Neurosurgery.

